# Women’s preferences for contraceptive counseling in Mexico: Results from a focus group study

**DOI:** 10.1186/s12978-018-0569-5

**Published:** 2018-07-16

**Authors:** Kelsey Holt, Icela Zavala, Ximena Quintero, Doroteo Mendoza, Marie C. McCormick, Christine Dehlendorf, Ellice Lieberman, Ana Langer

**Affiliations:** 1 Department of Social and Behavioral Sciences, Harvard T.H. Chan School of Public Health, Boston, USA; 2 Women and Health Initiative, Harvard T.H. Chan School of Public Health, Boston, USA; 3 Department of Family and Community Medicine, University of California, San Francisco, USA; 4 Program in Woman-Centered Contraception, University of California, San Francisco, USA; 5 Mexican Family Planning Foundation, Mexico City, Mexico; 6 Department of Epidemiology and Biostatistics, University of California, San Francisco, USA; 7 Department of Obstetrics, Gynecology, & Reproductive Sciences, University of California, San Francisco, USA; 8 Department of Epidemiology, Harvard T.H. Chan School of Public Health, Boston, USA; 9 Department of Pediatric Newborn Medicine, Brigham and Women’s Hospital, Harvard Medical School, Boston, USA

**Keywords:** Contraception, Family Planning, Patient Experience, Counseling, Latin America and the Caribbean, Qualitative Research Methods, Quality of Care

## Abstract

**Background:**

Client-centered contraceptive counseling is critical to meeting demand for contraception and protecting human rights. However, despite various efforts to optimize counseling, little is known outside of the United States about what individuals themselves value in counseling. In the present study we investigate women’s preferences for contraceptive counseling in Mexico to inform efforts to improve service quality.

**Methods:**

We conducted applied qualitative research, using six focus group discussions with 43 women in two cities in Mexico with distinct sizes and sociocultural contexts (Mexico City and Tepeji del Río, Hidalgo) to assess contraceptive counseling preferences. We used a framework approach to thematically code and analyze the transcriptions from focus groups.

**Results:**

Consistent with quality of care and human rights frameworks for family planning service delivery, participants expressed a desire for privacy, confidentiality, informed choice, and respectful treatment. They expanded on usual concepts of respectful care within family planning to include avoidance of sexual assault or harassment—in line with definitions of respectful care in maternal health. In contrast to counseling approaches with method effectiveness as the organizing principle, participants preferred counseling centered on personalized assessments of needs and preferences. Many, particularly older, less educated women, highly valued hearing provider opinions about what method they should use, based on those personalized assessments. Participants highlighted the necessity of clinical assessments or physical exams to inform provider recommendations for appropriate methods. This desire was largely due to beliefs that more exhaustive medical exams could help prevent negative contraceptive outcomes perceived to be common, in particular expulsion of intra-uterine devices (IUDs), by identifying methods compatible with a woman's body. Trust in provider, built in various ways, was seen as essential to women's contraceptive needs being met.

**Conclusions:**

Findings shed light on under-represented perspectives of clients related to counseling preferences. They highlight specific avenues for service delivery improvement in Mexico to ensure clients experience privacy, confidentiality, informed choice, respectful treatment, and personalized counseling—including around reasons for higher IUD expulsion rates postpartum—during contraceptive visits. Findings suggest interventions to improve provider counseling should prioritize a focus on relationship-building to foster trust, and needs assessment skills to facilitate personalization of decision-making support without imposition of a provider's personal opinions. Trust is particularly important to address in family planning given historical abuses against women’s autonomy that may still influence perspectives on contraceptive programs. Findings can also be used to improve quantitative client experience measures.

## Resumen

**Antecedentes:**

La consejería sobre anticoncepción centrada en la usuaria es fundamental para satisfacer la demanda de métodos anticonceptivos y proteger los derechos humanos. Sin embargo, a pesar de los esfuerzos enfocados a optimizar la consejería, se tiene poco conocimiento sobre lo que es importante de este proceso para las usuarias fuera de los Estados Unidos. En el presente estudio, se investigaron las preferencias que tienen las mujeres con respecto de la consejería de métodos anticonceptivos en México, con el fin de mejorar la calidad del servicio.

**Método:**

Se realizó una investigación cualitativa aplicada, utilizando seis grupos focales con 43 mujeres de dos ciudades de México con distintos tamaños de población y contextos socioculturales (Cuidad de México y Tepeji del Río, Hidalgo) con el objetivo de conocer sus preferencias en la consejería sobre métodos anticonceptivos. Se utilizó un enfoque de marco de referencia para crear códigos y realizar el análisis cualitativo de las transcripciones de los grupos focales.

**Resultados:**

Las participantes, en congruencia con el marco de calidad y derechos humanos en los servicios de planificación familiar, expresaron su preferencia por elementos como la privacidad, confidencialidad, decisión informada y trato respetuoso. Se abordó el concepto de “atención respetuosa” en planificación familiar para evitar el abuso sexual o acoso, lo cual concuerda con las definiciones de la atención respetuosa en la salud materna. En contraste con los enfoques que abordan la efectividad anticonceptiva como un principio rector de la consejería, las participantes estaban más interesadas en que la consejería se centrara en una asesoría personalizada de sus necesidades y preferencias. Muchas de ellas, particularmente aquellas con mayor edad y/o menor nivel de escolaridad, consideran muy importante saber la opinión del prestador de servicios de salud sobre cuál método anticonceptivo deben usar, basado en la asesoría personalizada. Las participantes destacaron la necesidad de recibir asesoría médica o realizarse exámenes físicos para informar las recomendaciones del prestador. Se identificó que este deseo se debe en gran medida a que entre las mujeres existen creencias respecto a los efectos o incidentes que pudieran surgir con el uso de algunos métodos anticonceptivos, particularmente en la expulsión del DIU, asumiendo que muchos de éstos se podrían prevenir con revisiones médicas más exhaustivas para determinar el método más adecuado para cada mujer. La confianza en el prestador de salud se construía de distintas formas, y fue percibida como un aspecto esencial para satisfacer las necesidades de las mujeres con respecto a los métodos anticonceptivos.

**Conclusiones:**

Los hallazgos permiten visibilizar la subrepresentación de las expectativas de las usuarias con relación a sus preferencias durante la consejería. Se resaltan recomendaciones específicas para mejorar la prestación de servicios en México, para asegurar que las usuarias cuenten con privacidad, confidencialidad, decisión informada, trato respetuoso, y asesoría personalizada—incluyendo otorgar mayor información sobre las razones por las que ocurren las expulsiones de DIUs postparto—durante las consultas sobre la anticoncepción. Los hallazgos sugieren que las intervenciones de consejería deberían priorizar un enfoque en el que se construya una buena relación entre el prestador de servicios y la usuaria, donde se fomente la confianza, así como contribuir a desarrollar habilidades de las y los prestadores de servicios de salud para asesorar las necesidades de las usuarias con el fin de otorgar un apoyo individualizado en el proceso de toma de decisiones sobre la elección del método anticonceptivo sin la imposición de las opiniones personales del prestador. La confianza es particularmente importante durante una consejería en planificación familiar debido a los abusos cometidos históricamente contra la autonomía de mujeres, que aún pueden estar influyendo en las percepciones sobre los programas de anticoncepción. Los hallazgos también pueden usarse para mejorar la evaluación cuantitativa de las experiencias de las usuarias.

## Plain English summary

Making sure health care providers give women information about birth control that meets their individual needs is important to human rights and reproductive health. However, few investigations have been conducted to ascertain desirable aspects of counseling from the woman’s perspective. We conducted this study in Mexico to try and fill this gap in knowledge through six focus groups with 43 women in two cities. Results indicated that participants expected many of the things that are already considered optimal for counseling (privacy, confidentiality, information, respect), and that they were also concerned about avoiding sexual assault and harassment from providers during family planning visits. Of particular importance was the desire for the counseling to be tailored to their own needs and preferences, rather than structured around prioritizing information about the most effective methods of contraception. In addition, some participants wanted providers to help them decide which method to use. Direct and indirect experience with the expulsion of intra-uterine devices led some individuals to believe that counseling sessions should include a clinical exam to see whether methods are right for them before they start using them, though this is not necessary from a clinical point of view. Finally, participants described that trusting the provider was essential to finding the right contraceptive method. These findings provide specific avenues for improvement of contraceptive counseling in Mexico.

## Background

Client-centered contraceptive counseling is at the heart of high quality family planning service delivery [1, 2]. Receipt of quality counseling has been associated with use and continuation of contraception among family planning clients in diverse settings [3–7] and has the potential to encourage individuals to return to the health system if they are not satisfied with a particular method [2]. Because concerns regarding side effects and health risks play a significant role in non-use of contraception among women not desiring pregnancy [8], good counseling to ensure women identify a method they are comfortable with and less likely to discontinue due to dissatisfaction is very important. Ensuring provision of high quality counseling is critical to meeting demand for contraception at an individual level, and reducing rates of undesired pregnancy at a population level. Further, high quality counseling is essential to promote informed decision-making, a critical component of human rights-based contraceptive service delivery [9].

Concerted efforts have been made to monitor quality of care from the client’s perspective since the early 1990’s in the global family planning field [2, 10], with typical survey questions probing about what information clients received and how they perceived treatment by the provider [10, 11]. However, despite this focus on measuring client experience and numerous efforts to optimize client experience through provider-facing or client-facing counseling interventions [12–17], very little is known about what individuals themselves value related to receiving counseling and decision-making support from family planning providers. Evidence from the United States (US) suggests clients desire personalized attention from contraceptive providers [18], and that—while there is a clear desire for autonomy in decision-making—many individuals value having their provider’s opinions about what methods might work well for them based on listening to their needs and preferences, in line with a shared decision-making approach [19, 20]. Evidence is lacking as to whether women in other settings share similar perspectives or instead prefer more or less involvement from providers in decision-making.

In the present study we investigate preferences for contraceptive counseling in Mexico, where 55% of pregnancies are not planned (this number rises to 70% in Mexico City) [21]. The national family planning program in Mexico guarantees provision of contraception without cost in the public sector, and its current strategic plan has identified the need for a focus on quality of care [22]. A recent study found deficiencies in interpersonal quality of care reported by a nationally representative sample of contraceptive clients in Mexico, particularly among adolescents, and corroborated the need for quality improvement efforts [23]. However, data on women’s preferences for contraceptive counseling in Mexico are needed to guide such efforts.

Counseling approaches can be optimized with better knowledge of what women value in their communication with providers. We sought to contribute to an international evidence base on client preferences for contraceptive counseling through this study among contraception clients in Mexico. This study was conducted as part of a larger project to construct the Quality of Contraceptive Counseling Scale for measurement of women’s perceptions of the quality of contraceptive counseling in Mexico, India, and Ethiopia. Results will be used to guide the development of the scale’s item pool.

## Methods

### Study design

To elicit women’s preferences for contraceptive counseling, we conducted applied research using focus group discussions (FGDs). The group interview setting of an FGD allows for individuals to share and consider their own experiences and thoughts in the context of others’ experiences and creates space for shared perceptions to emerge. Individual responses, as well as the interactions between participants, can be analyzed to identify areas of agreement about a topic [14, 16].

The Harvard T.H. Chan School of Public Health Institutional Review Board granted approval for the study after we obtained permission from a three-member community advisory board assembled for this study in Mexico.

### Population

Our recruitment sites were selected purposively to capture a diverse sample of urban contraception clients in Mexico. FGDs took place in Mexico City, the capital of Mexico, and Tepeji del Río. These two cities are very different sizes and have different sociocultural contexts. Mexico City is the economic and cultural center of Mexico with the most progressive policies in the country—including legalized abortion and same-sex marriage—and approximately nine million residents. In contrast, Tepeji del Río is a much smaller city of approximately 100,000 residents in the state of Hidalgo which has a substantially higher proportion of its population living in poverty compared to Mexico City (54% versus 28% in 2014) [24].

We also maximized diversity in terms of socio-economic status by recruiting in a public clinic in Mexico City serving a low-income clientele with no cost contraceptive services and private clinics in both cities operated by Mexfam, Mexico’s International Planned Parenthood Federation affiliate, serving a slightly more affluent population with low-cost contraceptive services. We also recruited women in a public area at a public university in Mexico City to ensure inclusion of university-aged women.

### Recruitment

Recruitment occurred through a combination of flyer distribution and in-person recruitment in waiting rooms and, in the case of women recruited at the university, in a booth in a public space. For in-person recruitment, participants who appeared to be of reproductive age were approached and invited to participate in a study to discuss their experiences talking with health care providers about contraception. They were told that the study’s findings would help inform efforts to improve healthcare service delivery.

Eligibility criteria included being female and age 18 or over, and having consulted any type of health care provider about contraceptive options at some point in the last three years.

Interested individuals were assigned to a focus group based on where they were recruited (i.e., public clinic, private clinic, or university setting), and focus groups took place at those same locations. Target enrollment in each focus group was 6-10 women. Each participant provided informed consent before participating in the FGD.

### Study procedures and instrument

After reviewing the IRB-approved consent form and providing verbal consent, each participant provided basic demographic information—including their age, occupation, and education level—to the study team. FGDs lasted two hours each and two female social science researchers (IZ, master’s level trained; and XQ, bachelor’s level trained) moderated and took notes during each discussion and prepared field notes after each discussion.

The semi-structured FGD guide focused on perspectives related to what constitutes an ideal interaction about contraception with providers, using the questions: “what makes the difference between a good and not-so-good interaction with a healthcare provider about contraception,” and “describe an ideal interaction,” with probes for what information should be provided by the provider, what information should be solicited from the client, and who should make the decision about which method to use. Moderators clarified at the beginning of each group that “provider” referred to any type of health care provider with whom they may speak about contraception, including doctors, nurses, counselors, psychologists, and social workers. The FGD guide also contained questions to explore the meaning to women of specific concepts central to quality of care and human rights when they did not spontaneously arise: confidentiality, privacy, empathy, respect, non-discrimination, and trust. These concepts were identified from the Quality of Contraceptive Counseling Framework[25]. The guide probed to understand to what extent these are prioritized by clients and to understand in more concrete terms what actualization of these concepts would look like to them.

### Analysis

FGDs were recorded and recordings transcribed verbatim in Spanish. In line with a Framework Approach to analysis of qualitative data [26], we began thematic analysis of transcripts with a list of a priori codes closely aligned with questions from the FGD guide (e.g., meaning of trust, expectations for information received, decision-making preferences) and developed additional codes inductively based on an initial review of all transcripts. After discussion among KH, IZ, and XQ and agreement by all on a final set of codes, all transcripts were coded by one of these three coders using the final code book and the software package Atlas.ti 7. Each coded transcript was reviewed by another member of the analysis team and discrepancies were discussed and resolved. After this initial phase of analysis, summaries of related groups of codes were developed to create a synthesized description of women’s preferences for care. All analysis was done in Spanish. Direct quotes are translated to English in the Results section.

## Results

### Participant characteristics

Forty-three women participated in a total of six FGDs between October and December 2015. Four groups were held in Mexico City and two were held in Tepeji del Río. Due to higher numbers of women in the Tepeji del Río groups, the number of participants from each city was comparable (24 in Mexico City versus 19 in Tepeji del Río) (Table 1). Three groups were held in Mexfam clinics, two in public clinics, and one in the university. The median age of participants was 25 and the majority had high school education or less and were not employed outside of the home (Table 1).

**Table 1:** Focus group participant characteristics (*N* = 43)

Characteristic	
Where recruited *n(%)*	
Mexfam clinic, Tepeji del Río (2 groups held)	19 (44%)
Public clinic, Mexico City (2 groups held)	11 (26%)
University, Mexico City (1 group held)	9 (21%)
Mexfam clinic, Mexico City (1 group held)	4 (9%)
Age *median(range)*	25 (18-60)^a^
Occupation *n(%)*	
Household work	26 (60%)
Student	12 (28%)
Employed outside of the home	5 (12%)
Education level *n(%)*	
Advanced (university, in progress or complete)	18 (42%)
Intermediate (high school or technical degree)	9 (21%)
Basic (middle school or less)	16 (37%)

^a^*Note* Despite our best attempts to screen participants and ensure they met our inclusion criteria of recent contact with a health care provider about contraception options, a few women not of reproductive age were allowed to participate after coming to FGDs with family members. These women participated in the discussion, but their individual quotes and opinions are not highlighted in the findings as they fall outside the target age range

### Women’s preferences for contraceptive counseling

Findings are grouped according to the Quality of Contraceptive Counseling Framework which distinguishes between relationship building and the steps in a counseling process that are influenced by the relationship between clients and providers [25].

#### Establishing trust

The concept of trust in the provider was at the heart of how women described an ideal interaction around contraception. Without trust, women consistently described feeling reluctant to express needs and preferences, or ask questions, and they highlighted lack of trust as a driving factor in deciding not to return to that same provider or institution in the future. Lack of trust was also often a reason for ending a visit without their needs for contraception met. Critical elements for establishing trust included ensuring privacy and respectful treatment upon initial interaction with the provider. Subsequent conversations and engagement, or lack thereof, of women in the discussion about contraception—including asking them what their concerns or motivations are—were described as continuing to influence the trust generated. This common theme about the importance of trust beginning early on in the consultation was clearly expressed by a 42-year old domestic worker in Mexico City:

“The image that the provider projects is important…In terms of generating trust …The way in which they look at you and they speak to you…you have to look for the type of person who is ready to serve, since they are going to have a [woman seeking contraception] who is probably passing through a difficult time in their life. This is what makes you feel trust…and makes you say ‘well I am going to return.’”

In elaborating on what providers should do to gain women’s trust, participants described the importance of exuding a positive, friendly, committed attitude, described by one person as an “attitude of service,” and to demonstrate that they are paying attention to women through eye contact and other body language. Several felt that addressing women fondly and warmly helped generate trust while others expressed that this was not a pre-requisite for establishing trust. One woman who was a 24-year old housewife with a high school education in Mexico City said that providers should be:

“Friendly so that trust is built and we have the confidence to ask them things. Because when you get a doctor who is a real grouch, you don’t feel like even asking them anything anymore and you just say ‘yes’ to them even though you have questions or you don’t even know how contraception methods work.”

Participants also desired a provider who displays confidence in his or her knowledge of contraception options; women in several groups discussed the lack of trust they feel when they observe providers hesitating or giving unclear or insufficient answers. One university student from Mexico City stated: “If you are seen by someone who is well informed, they give you confidence in being able to entrust something so, so precious as the control of your body [to them].”

#### Respectful treatment

The concept of respect was commonly evoked in discussions of what providers can do to make women feel comfortable and empowered to ask questions. Women commonly described respect in terms of respect for their *bodies* and respect for their *decisions*. Respect for women’s bodies was referred to in the sense that women expected providers to not make rude or judgmental comments about their bodies, and to not engage in inappropriate touching. When touching is necessary, participants expressed that the reason should be clearly explained before and during the process. Related to privacy, participants also expressed that others should not be allowed in the room during physical examinations (and if it is necessary, permission of the woman must be given). Many women expressed feeling much more comfortable and less embarrassed with female providers than male providers, particularly in cases where physical examinations or procedures were necessary, because they felt that females could be more empathetic given that they are “speaking the same language” and because, in some cases, male doctors can show an inappropriate interest in their bodies. However, others said that the gender of the provider was not as important as the way they treat you and make you feel comfortable, and pointed out that female providers can in some cases make you feel just as uncomfortable as a male provider.

Desire for feeling respected related to decision-making was discussed in regard to whether or not a woman chooses to use a method and, when she does, her decision about *which* method to use. According to participants, valuing a woman’s role in the decision-making process would be displayed by listening carefully to women, communicating in a professional way that does not involve mocking, shouting, or scolding, and not forcing her to use a method she does not want to use. For example, a university student from Mexico City explained respect as, “the fact that they are not saying to you, ‘oh, how bad, you are very young’ [referring to a young woman asking for contraceptives], but simply respecting your decision and giving you what you want.”

Non-discrimination also emerged in discussions of respectful treatment without prompting, with women describing not wanting to be judged by providers and asked extraneous questions or given extraneous information about what they should or should not be doing (e.g., related to religious beliefs or appropriateness of childbearing at certain ages) when asking for contraception information and options, just because of who they are. Possible discrimination was expressed mostly in relation to age. All groups discussed a concern for younger women being judged or scolded for having pre-marital sex. Less commonly, participants mentioned that older women receive less attention from providers or are judged for their desire to continue having children, and that discrimination can also occur based on sexual orientation or looks (looks in terms of both socio-economic status and attractiveness).

#### Privacy and confidentiality

When probing for the importance and meaning of privacy and confidentiality, groups’ discussions generally blended the two concepts into one. Women commonly emphasized the need for a private physical space and that information shared in the discussion should not leave the room without a woman’s consent. As stated by a 37-year-old housewife from Tepeji del Río, “It should be as if they [providers] guarded a secret. They shouldn’t be divulging the information to anyone besides the chart.” Several groups discussed and agreed that—while it is sometimes necessary for medical professionals to share information with other colleagues in order to ensure good quality treatment (e.g., if there is a concern for contraindications with other medications a person is taking) or for teaching purposes—one’s personal identification should be kept confidential.

An effect of not perceiving privacy is that one might not feel comfortable disclosing her needs to a provider if she thinks others can hear; one 20-year-old university student in Mexico City gave a personal example of having gone to a clinic as a young woman for the first time, hoping to ask for information about contraception options. Her interaction with the nurse was within earshot of the waiting room which resulted in her being too embarrassed to ask for information; she simply told the nurse she wanted some condoms, took them, and then had not returned since to ask for contraception.

Women discussed several ways providers can ensure a feeling of privacy, including explaining to women that it is a private discussion and that their information will be kept confidential, and closing the door.

#### Information exchange and decision-making support

A desire for clear, complete, and correct information was consistently communicated as the most important aspect of an ideal contraception counseling interaction. However, an important precursor to information provision, discussed in all groups, is the relationship building described in the previous sections. Women felt that, once trust is established, and if it is maintained throughout the discussion of contraception, the information exchange and decision-making process will be functional and fruitful.

Women described a desire for step-by-step descriptions of how contraception methods work and how they should be used, in plain language. The specific information groups consistently desired was what the expected impact is on a woman’s body, what the advantages and disadvantages (including contraindications) are, how effective the method is, how long each method can be used and when return visits are required, and what to do if a woman wishes to change or discontinue a method. For example, as one woman (age and occupation unknown) from Tepeji del Río explained:

“The ideal is that before you start a method you know all of the causes, benefits, and consequences, and that the doctor explains them to you. Because a person probably has information but it’s not correct, or it’s myths.”

Women’s descriptions of the ideal information exchange and decision-making process matched closely with the idea that counseling should be individualized based on listening to an assessment of needs, preferences, prior experiences, and medical history. Groups diverged somewhat based on the age composition in their vision for whether women should receive complete information about *all* contraception options. Younger women under the age of approximately 25 (particularly in the group of university students) conveyed more of a desire to be fully informed about all options. Older women expected the provider to play a more central role in selecting a method for a woman to then approve of (or not), regardless of whether all method choices are mentioned. Across all groups, a common expectation was that the provider has a role to play in recommending particular methods to women, but that the woman then should have the ultimate power over decisions about whether to use that method (or any method). This process was referred to as a decision made “by two” in which the ideal is to reach a point of agreement about which method to use and in the case that an agreement cannot be reached, the provider must trust the woman’s decision. For example, a university student from Mexico City answered a question about who should make the decision about a woman’s contraceptive choice by saying,

“It should be between both [woman and provider], no? In other words, the doctor should tell you, ‘this one suits you because of this, this, and this,’ and you would say, ‘but I prefer this one, I feel more comfortable with this one.’ In other words, a decision made by both.”

It was very clear in the groups that, even when a woman appreciates the provider making an initial suggestion about which method she should use, she or he should not force the woman to use that method or refuse to remove it in the case that she changes her mind.

The most commonly discussed factor women thought should influence the recommendation a provider makes about an ideal contraception method for a woman was physical compatibility between a method and a woman’s unique body; the phrases “appropriate for your body” and “fits your body” were used multiple times in different groups to describe this sentiment. Behind this was a widely held understanding of women’s bodies as having the potential for different reactions to the same contraception method based on hormonal, menstrual, and physical (e.g., cervical/uterine characteristics) factors that providers should review in order to be able to give women advice about the potential for a method to cause harm or be “rejected” by the body. A 27 year old woman from Tepeji del Río said:

“I would like for them [the provider] to tell me, ‘you know what, look, the IUD [intra-uterine device] is for this and this,’ in other words ‘the consequences you will have are these.’ The pros and cons. Also upon giving me a physical exam if I have something in my cervix or something to say, ‘you know what, you can’t use this contraceptive because it will harm a certain part of your body.’ Or, ‘you know what, use the patch but not the IUD because it will harm…your cervix’ or whatever. But if they give me the physical exam and tell me I’m fine, well, then bring on all of the contraceptive methods. But they should give me the follow-up, like, ‘you can use this because you don’t have any risks.’”

Many women either had experienced postpartum IUD expulsion or knew others who had. This appeared to be a key driver of a belief in the need for a physical exam before determining if a method would be “rejected” by one’s body.

Relatedly, some participants pointed to health factors that can increase a woman’s risk for complications from a method, including factors related to mood, weight, and allergies. They expected providers to evaluate these and explain potential side effects and complications to women when telling them a particular method is not right for them. Less commonly, women pointed out that lifestyle, personality, and sexual behavior also affect the assessment of an ideal method for a woman and should therefore be taken into account by providers when making a recommendation. All groups agreed that providers should recommend a method after weighing pros and cons considering a woman’s particular situation and body. Specifically, they felt providers should not make biased recommendations based on assumptions about what is best for a woman because of her age, number of children, or other characteristics. The commonly used phrases were “suits you,” “compatible,” and “the most adequate,” to describe methods providers should recommend.

When describing how this decision-making process should work, women often evoked an ideal of feeling understood, taken seriously, and included in the process by being asked directly about their preferences. One 24 year old university student from Mexico City stated:

“We need personalized treatment because contraceptive methods are always spoken of in general terms but you are never included in the plan, like, ‘What would you like to use? What would be best for you?’. It’s always like they tell you, ‘these you can use,’ but they never ask your opinion to know if they’ll work for you or not…your experiences, so that they can help you to find a method that will be useful for you.”

Women suggested providers actively engage women by asking what their preferences are and whether they feel comfortable with the recommended method(s), and by reserving ample time for questions and doubts to be expressed and resolved. The idea of taking concerns seriously and patiently, and clearly responding to them rather than evading them, came up in multiple groups. Some participants expressed a preference for providers proactively checking with them regularly to see if the method is working for them.

In several groups, women valued visual learning and preferred providers to show women method choices when offering options and also, as applicable, when describing in more detail how a particular method works (e.g., how an IUD is inserted or how a patch should be placed on the body). In one group, participants suggested complementing verbal and visual information with written information or referrals to internet sites.

#### Women’s contributions to the counseling process

In most groups, the idea that women themselves are also responsible for ensuring positive interactions with providers was discussed. Some participants expected that women seek outside information about contraception options before talking with a provider to be able to have in mind what method they might like to use and to be able to ask the right questions. This sentiment often emerged after descriptions of negative prior experiences with providers, demonstrating in some contexts a recognition that one cannot expect an interaction to go well and, thus, that they must prepare themselves to help steer the conversation in a productive way (as one woman put it – to “resist” the provider). On the other hand, this expectation was also grounded in a reluctance to place sole responsibility for an interaction going well on the provider.

## Discussion

Our focus group findings shed light on what contraceptive clients desire from their interactions with health care providers in two urban Mexico settings. In line with family planning quality and rights frameworks [2, 9], our participants spontaneously expressed an expectation that services be provided with privacy and confidentiality, that detailed information be provided, and that providers communicate with clients respectfully. Somewhat outside the scope of current quality and rights frameworks, participants also brought up the importance of making sure providers are not touching women in inappropriate ways or taking an inappropriate interest in their bodies during family planning visits. This expectation of avoidance of sexual assault and harassment is, however, in line with definitions of respectful maternity care which include the right to be free from physical abuse [27].

In line with recommendations from the World Health Organization related to personalization of counseling [28], we found a common desire for providers to engage with women in the decision-making process, tailoring information and recommendations based on a comprehensive assessment of women’s health status and preferences, rather than simply detailing each individual method and asking women to make their own decisions as in a more consumerist approach [29]. This is similar to research in the US which indicates a preference for personalized counseling in line with a shared decision-making approach [18–20], and contrasts with tiered counseling approaches which are organized around prioritization of the most highly effective methods rather than personal preferences [15, 17].

We noted a difference in expectations for information by age, whereby university-aged women tended to express a desire for complete information about method options to make autonomous decisions, while older women felt more comfortable with providers excluding information based on their assessment of what method would meet women’s needs. This age difference could be in part due to a cohort effect stemming from societal changes in Mexico in beliefs regarding reproductive autonomy brought about largely by the decriminalization of abortion in Mexico City in the last decade [30, 31], or an aging effect where women tend to value provider input more as they age. It is also important to note that the majority of the younger women were university educated (whereas the rest of the sample was not) and located in Mexico City. Thus, the noted age differences may also have to do with education level or city, as the cultural shift towards valuation of reproductive autonomy has been largely concentrated in Mexico City.

Gradations in client preferences for provider input into contraceptive method decision-making in our study suggest providers’ counseling approaches should be flexible to accommodate these preferences. In line with the human rights principle of informed choice in contraceptive programs [9], providers should continue to focus on provision of comprehensive information to support clients’ informed decision-making, without ruling out particular methods for clients based on the provider’s own preferences. In cases where patients indicate they value providers’ opinions, the personalized counseling approach—whereby providers ask a series of questions to elicit a client’s own preferences for particular method characteristics (i.e., bleeding changes, hormonal composition, effectiveness, mode of use, etc.)—can guide the provider to make a method recommendation based on client preferences rather than their own opinions.

Despite disagreements about the amount of information providers should give women about different method options, participants universally agreed that providers must ultimately respect the woman’s right to make a final decision about contraceptive use. This belief is in line with recommendations from the paradigm-shifting 1994 International Conference on Population and Development (ICPD) and more recently published human rights standards [9, 32]. Qualitative research with male providers in Mexico has identified paternalistic beliefs about female patients, which manifests in some cases as directive counseling and imposition of sterilization and IUDs [33]. In our study, participants described provider judgment of women’s sexual activity and judgmental and discriminatory behavior, particularly towards young women, as a key threat to good counseling. Counseling interventions should prioritize emphasis on respect for women’s choices and exclusion of providers’ personal opinions during decision support, recognizing and addressing the power differentials inherent in patient-provider relationships and the gender dynamics at play that can manifest as paternalistic beliefs.

Another common theme that emerged from FGDs was a belief in the necessity of clinical assessments, and in some cases physical exams, to assess compatibility of hormonal methods and IUDs. Assessment of medical eligibility criteria is indeed a critical part of contraceptive counseling, given that certain methods are not medically appropriate for women, given their age or health history. For example, women over age 35 who are smokers should not use combined hormonal contraception [28]. However, the preference expressed by women in our study for clinical assessments or physical exams was rooted in a belief that different women will have different reactions to the same method due to individual hormonal, menstrual, and/or cervical/uterine factors, and that tests or exams can help to prospectively identify these differences between women. This is not in line with best practices for contraceptive care [28]. Another qualitative study in Ghana with women recruited in clinics similarly revealed a belief that blood tests were necessary for clinicians to recommend ideal methods for individual women [34]. This suggests the need for careful counseling to validate and respectfully address women’s concerns about potential safety or fit issues, while at the same time not conducting unnecessary physical exams or blood tests. In our study, many women either had experienced postpartum IUD expulsion or knew others who had (unsurprisingly, given that post-partum IUD uptake is common in Mexico [35]), and this appeared to be a key driver of a belief in the need for a physical exam before determining if a method would be “rejected” by one’s body. Given this, it is of critical importance to make sure women are counseled around expulsion rates, the higher expulsion rate with postpartum IUDs, and the lack of ability to predict whether an individual person will experience this outcome. [36].

Women’s expressed preferences for contraceptive counseling consistently centered on the concept of trust—built on a foundation of respect—as paramount to facilitating good communication and ensuring women identify a method they are happy about and return for care if needed. Trust in health care providers is a known factor in health care seeking behavior generally [37], and is particularly important in the context of family planning where there have been historical abuses of women’s autonomy [38]. In Mexico, large investments in family planning programming began in the 1970’s but were motivated by demographic goals, drawing suspicion from feminist groups for the lack of attention to women’s needs [39]. Though family planning programming was later reframed in Mexico and elsewhere in terms of rights and empowerment, in line with the ICPD conference, the historical context of population control may still permeate some communities’ perspectives on contraceptive programs. Thus, concerted efforts to build clients’ trust, building on the longstanding recognition in the field that interpersonal relationships between providers and clients are a critical component of quality care [2], are essential to optimizing contraceptive counseling.

Our findings are limited by the fact that we recruited women in only two states and that the majority of participants were recruited in Mexfam clinics which serve a primarily middle class population. Further, because we specifically targeted university students in order to understand the specific contraceptive counseling preferences among this population, we ended up with a large percentage of our participants (42%) who were university educated. Because of these factors, we did not reach many women living in extreme poverty or indigenous women, and due to eligibility criteria, women under 18 years old were also not included. Future work to examine the applicability of these findings to other populations in Mexico is warranted. Additionally, because recruitment took place primarily in health care settings, it is possible that we excluded women who were not connected to the health care system, including those who no longer seek health care due to negative prior experiences. These women may have different preferences than those captured in this study. Finally, we did not include a question in the FGD guide asking groups to discuss preferences around involvement of sexual partners in contraceptive counseling. This is an important area for further study to inform counseling improvement efforts. Despite these limitations, this work is particularly timely for development of new approaches to improving counseling which could benefit from information on women’s preferences for relationship building, information provision, and decision support from contraception providers.

## Conclusions

The findings from this study are unique in shedding light on the under-represented perspective of what clients themselves desire from contraceptive counseling. Specific expectations described in this paper around what core concepts such as respect, informed choice, personalized counseling, privacy, and confidentiality look like to women in more concrete terms can be used to bolster quality improvement efforts and measurement of client experience in urban Mexico. The common misunderstanding that clinical or physical examinations are required for determining one’s personal eligibility for contraceptive methods can be addressed through better counseling around postpartum IUD expulsion rates and acknowledging that women have variable responses to contraceptive methods that cannot be determined prospectively. The centrality of trust and respect to women’s descriptions of high quality counseling drive home the need for emphasizing the ability to elicit and address women’s specific needs, preferences and concerns, as primary skills for any contraceptive provider.

## Abbreviations

FGD: Focus group discussion
ICPD: International Conference on Population and Development
IUD: Intra-uterine device
US: United States
